# A Novel Recombinant Vitronectin Variant Supports the Expansion and Differentiation of Pluripotent Stem Cells in Defined Animal-Free Workflows

**DOI:** 10.3390/cells13181566

**Published:** 2024-09-17

**Authors:** Xi Lu, Eli Perr, Tahmina Naqvi, David Galitz, Marnelle Andersen, David Grabowski, Anthony Person, Alex Kalyuzhny, Kevin C. Flynn

**Affiliations:** 1 Stem Cell & Gene Therapy, Bio-Techne, Minneapolis, MN 55413, USA; xi.lu@bio-techne.com (X.L.); tigerperr@gmail.com (E.P.); tahmina.naqvi@bio-techne.com (T.N.); dsgalitz@gmail.com (D.G.); marnelle.andersen@bio-techne.com (M.A.); 2 Protein Development, Bio-Techne, Minneapolis, MN 55413, USA; david.grabowski@bio-techne.com (D.G.); anthony.person@bio-techne.com (A.P.); 3 Antibody Applications, Bio-Techne, Minneapolis, MN 55413, USA; kalyu001@umn.edu; 4 Department of Biochemistry & Molecular Biology, Colorado State University, Fort Collins, CO 80523, USA

**Keywords:** vitronectin, pluripotent stem cells, animal-free, substrate

## Abstract

An essential aspect of harnessing the potential of pluripotent stem cells (PSCs) and their derivatives for regenerative medicine is the development of animal-free and chemically defined conditions for ex vivo cultivation. PSCs, including embryonic and induced PSCs (iPSCs), are in the early stages of clinical trials for various indications, including degenerative diseases and traumatic injury. A key step in the workflows generating these cells for more widespread clinical use is their safe and robust ex vivo cultivation. This entails optimization of cell culture media and substrates that are safe and consistent while maintaining robust functionality. Here, we describe the design of a human vitronectin (hVTN) variant with improved manufacturability in a bacterial expression system along with improved function in comparison to wild-type VTN and other previously characterized polypeptide fragments. In conjunction with an animal component-free media formulation, our hVTN fragment provides animal-free conditions for the enhanced expansion of iPSCs. This hVTN variant also supports the reprogramming of PBMCs into iPSCs. Furthermore, we show that these iPSCs can be efficiently differentiated into the three major germ layers and cortical neurons, thereby closing the loop on a completely defined animal-free workflow for cell types relevant for regenerative medicine.

## 1. Introduction

Human-induced pluripotent stem cells (iPSCs) act similarly to embryonic stem cells (ESC), possess almost unlimited expansion potential, and retain the capacity to differentiate into any adult cell type in the body [1,2,3,4]. These qualities make them invaluable as the basis of models for developmental diseases [5], drug discovery [6], drug delivery [7], and cell therapies in regenerative medicine [8]. Differentiated iPSCs are being investigated for use in multiple cell replacement therapies, including degenerative diseases such as Alzheimer’s [9,10] and Parkinson’s [11,12] as well as cardiovascular disorders [13,14], and are being evaluated for immunotherapy including induced Natural Killer cells [15,16]. Human iPSC-derived organoids and model systems have been used in toxicology studies for familial dysautonomia [17], long QT syndrome [18], and spinal muscular atrophy [19]. iPSC-derived intestinal [20], liver [21], brain [15], and cardiac organoids [13] have been utilized for oncological studies [22], personalized medicine [23], and even for understanding human evolution [24]. In addition, exosomes derived from differentiated iPSCs have been studied and shown to support tissue regeneration [25,26,27].

To fully realize the potential of iPSCs for regenerative medicine, animal-free expansion systems, including media and substrates, are required in order to not only increase experimental and manufacturing reproducibility but also limit the spread of potential animal contaminants [28]. For feeder-free expansion, iPSCs have previously been cultured on basement membrane extract (BME, e.g., Cultrex Ultimatrix and Matrigel) [29]. This substrate was originally derived from an Engelbreth–Holm–Swarm mouse sarcoma and is composed of four key structural proteins including collagen IV, laminin, entactin, and heparan sulfate proteoglycans [30]. However, there is significant lot-to-lot variability with BME, as it also contains more than a thousand other proteins, including transcription factors and cytokines; even growth factor-reduced versions of the matrix can still contain trace presence of these factors [31]. While other more defined extracellular matrix (ECM) proteins such as Vitronectin (VTN) [32,33], laminin (LN) [34,35], and peptides [36] have been used to culture iPSCs, manufacturing these proteins at scale using an animal-free process is a critical consideration. VTN is a 54 kDa glycoprotein commonly found in the extracellular matrix (ECM) and serum; it is known to promote cellular adhesion and spreading, including for iPSCs via recruitment of integrin receptors such as αvβ5 [37]. While a complete high-resolution structure has not been established for VTN, it is typically divided into three regions: the N-terminal Somatomedin B, central domains with hemopexin homology, and a C-terminal heparin binding domain [38]. It has been previously shown that vitronectin fragments with just the hemopexin domain and an RGD motif are capable of supporting iPSCs adhesion [39]. However, these VTN fragments are not always manufactured efficiently or using an animal-free process, despite being expressed with *E. coli* [32]. In this report, we describe the design and production of a soluble animal-free VTN fragment, VTN62-292, that can be manufactured in higher yields. In addition, VTN62-292 supports reprogramming and long-term culture of iPSCs better than full-length VTN or other VTN variants without compromising pluripotency or generating genomic instability.

## 2. Materials and Methods

Construction and expression of protein fragments: The work described here is covered in U.S. Provisional Patent Application No. 63/239,456. Human VTN was originally amplified using PCR essentially as described and cloned into a pET *E. coli* T7 expression vector with a C-terminal His tag [40]. The smaller VTN variants were constructed from this full-length VTN vector using site-specific primers and PCR amplification from the original vector and subcloning into the T7 vector. Animal component-free expression of the VTN variants was performed in shaker flasks with BL21 *E. coli* cells.

Preparation of Coated Substrates: Tissue culture surfaces were coated with either Cultrex Ultimatrix (R&D Systems by Bio-Techne, Minneapolis, MN, USA) or Vitronectin fragments. Briefly, surfaces were incubated with Ultimatrix diluted in cold buffer (1X PBS or DMEM) at 1:100 for at least 30 min at 37 °C or overnight at 4 °C. Purified Vitronectin fragments were diluted in PBS and incubated either overnight at 4 °C or at least 60 min at 37 °C. Coating solutions were aspirated just prior to cell seeding.

Cell Culture: Human iPSC lines used in this work, including 12-10a (Cell Applications, Inc., San Diego, CA, USA), BXS0114 (ATCC, Manassas, VA, USA) and BYS0110 (ATCC), were maintained in an animal origin-free Excellerate iPSC Expansion medium (R&D Systems by Bio-Techne, Minneapolis, MN, USA) in a humidified incubator with 5% CO_2_ with atmospheric oxygen at 37 °C. Cells were fed daily and routinely passaged as clumps by incubation with Versene (Thermo Fisher Scientific, Waltham, MA, USA) at 70–80% confluency at room temperature for 5–7 min. The dissociation medium was aspirated, then iPSC Expansion medium was added to lift the cells and split to a new plate. For single-cell dissociation, cells were treated with Accutase (Thermo Fisher Scientific) for 5–7 min at room temperature to obtain single cells and then collected after spinning down at 1000 RPM for 4 min. For the first 24 h, after harvesting with Accutase, cells were cultured with 5 µM of a ROCK-inhibitor (Y27632) and then fed daily with fresh iPSC medium. Live cell counts were performed using a ViCell analyzer (Beckman Coulter, Brea, CA, USA). Live Cell imaging was performed for measuring cell growth through confluence measurements using an Incucyte^®^ Live-Cell Analysis system (Sartorius, Gottingen, Germany). In these experiments, 12,000 cells/cm^2^ were plated in 24-well plates and imaged every 60 min for up to 4 days.

Adhesion assays: iPSC adhesion was assessed using a modified cell adhesion assay based on previously published methods [41,42]. Briefly, iPSCs were plated at 72,000 cells/cm^2^ in 96 well plates at varying concentrations of VTN protein. Poly-L-lysine (R&D Systems by Bio-Techne, Minneapolis, MN, USA) and Ultimatrix BME were used as non-VTN positive controls. Cells were allowed to settle and adhere for 20 and 60 min. The wells were washed by manual pipetting in PBS and forcibly inverted to remove non-adherent cells. CellTiter-Glo 2.0 (Promega, Madison, WI, USA) was used to detect adherent cells with luminescence.

Flow Cytometry: Differentiated and undifferentiated iPSCs were harvested using Accutase. An H/M Pluripotent Stem Cell Multi-Color Flow Cytometry Kit was used to identify stemness markers. For differentiation to different germ layers, we used antibodies including SOX17 (R&D Systems, Minneapolis, MN, USA, Catalog#: IC1924A), OTX2 (R&D Systems, Catalog#:AF1979), Brachyury (R&D Systems, Catalog#: IC2085A) at 5 μg/mL. Flow cytometry was performed using the LSR Fortessa instrument (Becton Dickenson, Franklin Lakes, NJ, USA) and analyzed in Flowjo software (Flowjo LLC, Ashland, OR, USA, v10.8). A minimum of 10,000 cells were used in each flow run. Gating was determined based on isotype controls. In some experiments, Mean Fluorescent Intensity (MFI) was used for comparisons. In other experiments, the percentage of cells expressing a particular marker was calculated based on a threshold intensity determined by gating on the isotype control intensity.

Reprogramming: Feeder-free reprogramming was performed using a Cytotune Sendai 2.0 kit (Thermo Fischer Scientific) and peripheral blood mononuclear cells, following the manufacturer’s instructions with some minor modifications; specifically, following the transduction step, cells were plated on VTN62-292 and transitioned into ExCellerate Media. Newly formed iPSC colonies were cloned and banked at various time points following the transduction step.

Pluripotency: Human iPSCs were expanded to confluency with daily media exchanges for iPSC maintenance media in 24-well plates coated with 5 μg/mL VTN62-292 or 1:100 Ultimatrix as a control. Cells were differentiated to the three germ layers (endoderm, mesoderm, and ectoderm) as a baseline measure of pluripotency. The Human Pluripotent Stem Cell Functional Identification Kit (Catalog#: SC027B) was used per the manufacturer’s instructions for ectoderm and endoderm, while 5 μM CHIR99021 (Tocris^®^ Bioscience by Bio-Techne, Minneapolis MN, USA) was added to the media for two days for mesoderm. The base media for all three germ layers differentiations was RPMI-1640 with Glutamax and N21 Max Insulin Free (R&D Systems by Bio-Techne, Minneapolis, MN, USA). Cardiomyocyte and hepatocyte differentiations were performed using the StemXVivo Cardiomyocyte kit (Catalog#: SC032B) and StemXVivo Hepatocyte kit (Catalog#: SC033), respectively, with animal-free N21 supplements (R&D Systems by Bio-Techne, Minneapolis, MN, USA).

Animal Component Free Neuronal Differentiations: Cells were differentiated into forebrain neurons in a manner similar to previous studies [43] except that all reagents were animal component-free. After at least five passages in animal component-free ExCellerate iPSCs media and VTN62-292, 12-10a and BXS0114 cells were replated at high density on 5 μg/μL VTN62-292 with GMP N-2 supplement (R&D Systems by Bio-Techne, Minneapolis, MN, USA) and ascorbic acid (0.2 mM). We used 5 μM Y-27632 GMP (Tocris^®^ Bioscience by Bio-Techne, Minneapolis MN, USA) in the first 10–12 h to promote cell survival. Neuronal specification was achieved with the combination of LDN 193189 GMP and SB 431542 GMP (both from Tocris^®^ Bioscience by Bio-Techne, Minneapolis, MN, USA) over 12 days in culture. At this stage, neural progenitor cells (NPCs) were passaged and transitioned into neuronal media with GMP BDNF (R&D Systems by Bio-Techne, Minneapolis, MN, USA) and an animal component-free N21 MAX supplement for an additional 20 days. Differentiated neurons were detected with beta III tubulin (TUJ1) and NeuN. Intermediate NPC differentiation was assessed with immunostaining for PAX6 and SOX1. Quantifications of neuronal differentiation were assessed using the Operetta high-content analysis system (Perkin Elmer, Waltham, MA, USA). DAPI staining was used to identify all cells in the culture, and neurons were identified with positive TUJ1 staining set at an empirically determined threshold based on intensity measurements of positive cells compared to controls (isotype and secondary control staining). The percent positive TUJ1 cells was percented to indicate neuronal development.

Immunocytochemistry: Cells were fixed with 4% Paraformaldehyde for 15–20 min and washed three times with 1 X PBS. Then, cells were blocked with 10% Donkey Serum in 1% Bovine Serum Albumin/PBS and 0.3% Triton-x-100 for 1 h. Cells were stained with primary antibodies overnight at 4 °C and washed three times with PBS. Samples were then incubated with secondary antibodies for 1 h, washed three times with PBS, and labeled with DAPI for 3 min. Finally, cells were washed three times and then stored in PBS at 4 °C in dark. The primary antibodies used were OCT3 (R&D Systems, Catalog#: AF1759), NANOG (R&D Systems, Catalog#: AF1779), TRA-1-60 (R&D Systems, Catalog#: MAB4770), SOX17 (R&D Systems, Catalog#: AF1924), OTX2 (R&D Systems, Catalog#: AF1979), Brachyury (R&D Systems, Catalog#: AF2085), NeuN (Novus, Catalog#: NBP1-77686), PAX6 (R&D Systems, Catalog#: AF8150), SOX1 (R&D Systems, Catalog#: AF3369), and Beta III tubulin (R&D Systems, Catalog#: AF3369). Primary antibody concentrations were used at 10 µg/mL unless otherwise indicated.

Statistical Analysis: Values are presented as mean ± SD unless otherwise specified Statistical significance was assessed using multivariate ANOVA in Graphpad Prism 10.1.2. Significance was held at *p* < 0.05.

## 3. Results

### 3.1. Design and Construction of Vitronectin Variants

Several critical findings inform the design of VTN variants. VTN is synthesized as a polypeptide that is 478 amino acids in length with a molecular weight of 75 kDa, with glycosylation accounting for about 30% of the mass [44]. In addition to a signal sequence with 19 amino acids, the functional VTN domains include somatomedin (SMB), hemopexin (Hp4), and heparin binding (Hb). Adjacent to the SMB domain is the integrin-binding Arg-Gly-Asp (RGD) motif, which is essential for its ability to act as a substrate for various cell types in cell culture. Previous work has shown that removing the N-Terminal SMB domain alone (VTN-N or VTN62-478) or in conjunction with the C-terminal V10 domain (VTN-NC, or VTN62-398) results in a recombinant VTN variant that can support pluripotent stem cells in culture. Although these variants retain and even improve adhesion and survival bioactivity in culture compared to wildtype (VTN-WT), they result in insoluble protein when made in *E. coli*, which requires additional solubilization steps that complicate large-scale manufacturing [32]. We confirmed the low yields of soluble protein of these two VTN variants (Supplemental Figure S1A).

**Figure 1:**
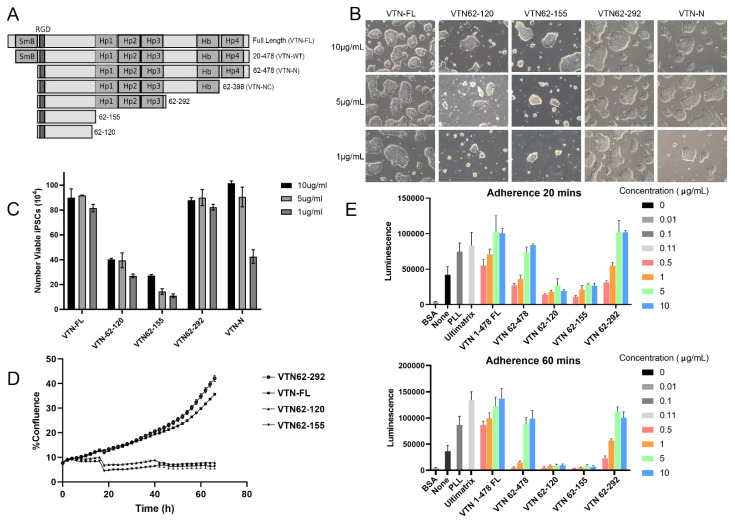
Screening vitronectin variants for iPSC cell culture. Schematic diagram of full-length VTN and different VTN fragments screened in this work (SMB = Somatomedin B domain; HP4 = hemopexin; Hb = Heparin binding) (**A**). Representative brightfield images of human iPSC (12-10a) cultured on different concentration of the VTN variants at 10, 5, and 1 µg/mL. All images were taken at 10× magnification. (**B**). Brightfield images show expected healthy morphology on VTN-FL and VTN62-292 at all concentrations. VTN-N supports iPSCs at all concentrations but shows noticeably less growth at 1 μg/mL. VTN62-155 and VTN62-120 support some reduced iPSC colony growth at 10 μg/mL, but the colonies round up and detach at the lower concentrations. The graph shows the number of viable iPSCs three days after seeding at the same initial density (**C**). The cell numbers (×10^4^) are shown from three experiments at the indicated concentrations. Note that VTN-FL and VTN62-292 support cell growth at all concentrations. Notably, VTN-N (VTN62-478) shows a decrease in cell number at 1 μg/mL compared to VTN62-292 (*p* ≤ 0.0003) (**C**). A graph of the percent confluence of iPSCs in cell culture over time from live-cell imaging experiments on the indicated VTN substrates at 5 µg/mL shows that VTN62-292 supports the growth of iPSCs as well as VTN-FL (**D**). Adhesion assays were performed with the VTN variants from 0–10 μg/mL with iPSCs (12-10a) at 20 min and 60 min (**E**). The adherent cells were measured with luminescence using a CellTiter-Glo assay. The results indicate that VTN62-292 supports iPSC adhesion nearly to the level of VTN-FL. Note that VTN62-292 has significantly higher adhesion than VTN 62-478 (VTN-N) at lower concentrations (e.g., *p* ≤ 0.01 at 1 μg/mL and 60 min). N = 3 for all experiments shown. Error bars indicate standard deviation.

The VTN variants produced and tested in this work retain the integrin-binding RGD site at amino acids 64–66, while the compact N-terminal somatomedin B (SmB) domain (at amino acids 20–63) was deleted in all with the exception of the wildtype VTN-WT (Figure 1A). Removal of the SmB domain supports human PSC attachment and survival better than wildtype [32], perhaps by allowing increased access to the RGD motif, which is critical for integrin recruitment and cell adhesion. In fact, an RGD peptide-grafted surface for culturing embryonic stem cells demonstrates that the RGD domain is the minimal component required for cell attachment and growth [45,46]. The heparin-binding (Hb) region (at amino acids 362–395) was present in several of the variants produced and tested here. The protein yields of these various constructs were tested in standard *E. coli* cultures for the heterologous expression of recombinant protein. While the expression of longer fragments up to amino acid 398 resulted in poor yields in *E. coli*, the VTN62-120, VTN62-155, and VTN62-292 variants were soluble and resulted in high expression (Supplemental Figure S1A). Full-length VTN (FL-VTN) was incompatible with animal-free production in *E. coli*; therefore, a full-length version (FL-VTN) made in animal cells was used for functional comparisons. A wildtype VTN with deletion of the N-terminal signal sequence, VTN20-478 (VTN-WT), is functionally identical to full-length VTN and can be expressed in an *E. coli* expression system [32]. VTN-WT is commercially available but requires additional purification from inclusion bodies. VTN fragments were expressed in *E. coli* and the purity was analyzed by SDS-PAGE and Coomassie Blue staining. The VTN variants all were purified as one major band at the expected molecular weights. Due to the enhanced performance of VTN62-292, as discussed below, we focused on optimizing the expression and purification of VTN62-292. A comparison of multiple independent lots of VTN variants made under animal-free conditions demonstrated that VTN62-292 shows improved yields of more than threefold compared to WT-VTN and more than twofold compared to VTN-NC (VTN62-398) (Supplemental Figure S1B). After extensive optimizations, purity of VTN62-292 at more than 95% was achieved (Supplemental Figure S1C). In the subsequent experiments, VTN-N (VTN62-478) was used instead of VTN-NC because it is commercially available and shows similar performance with PSCs [32].

### 3.2. Identification of Optimal Vitronectin Variant for Growth of iPSCs

To determine the range of concentration of VTN fragments necessary for supporting iPSC growth, iPSCs were cultured on 1, 5, and 10 µg/mL of different fragments (Figure 1B,C). VTN62-292 and VTN-FL were capable of supporting the attachment and expansion of iPSC colonies at all concentrations tested. The number of viable iPSCs obtained was similar between VTN62-292 and full-length VTN at all concentrations. The shorter VTN fragments, 62–120 and 62–155, had reduced ability to support growth of iPSCs at any of the tested concentrations (Figure 1B,C). Despite the attachment of a few cells that formed colonies, there was significant cell death for these short fragments at any concentration. Interestingly, while the commercially available recombinant VTN-N showed similar colony morphology and cell viability compared to VTN62-292 and VTN-FL at higher concentrations, it showed reduced cell numbers at 1 µg/mL. These data were confirmed in two additional iPSC cell lines which showed lower expansion at 1 µg/mL of VTN62-292 (Supplemental Figure S2). Live-cell imaging assays following iPSC cell confluence over four days in culture corroborated these findings (Figure 1D, Supplemental Video S1).

The differences in the ability for VTN variants to support iPSCs expansion have multiple possible mechanistic explanations, such as differences in cell adhesion, cell cycle regulation, and survival signal transduction pathways. We first tested the simplest explanation, namely, that these differences in cell growth are at least partly due to differences in the ability of these VTN variants to support initial cell adhesion. Using a modification of traditional adhesion assays [41,42], we tested the relative adhesion of iPSCs on different VTN variants. In this assay, cells were seeded onto the appropriate substrate and allowed to adhere for 20 or 60 min; non-adherent cells were forcibly washed off, and the remaining adherent cells were quantified via CellTiterGlo. At both time points, VTN62-292 showed significantly better cell adhesion than other truncation mutants and similar cell adhesion as full length VTN (Figure 1E). One hour after cell plating, VTN62-292 showed higher adhesion than all other variants with the exception of the FL-VTN. The shorter fragments, VTN62-120 and VTN62-155, showed lower adhesion at all concentrations at 20 min with decreased signal after 1 h, suggesting that these fragments support initial attachment but not sustained adhesion. The binding of iPSCs to VTN62-292 was also compared to the VTN-N variant, and showed similar adhesion at 20 min and 60 min (Supplemental Figure S2A). These static adhesion assays were confirmed with live imaging assays which were performed with imaging before and after vigorous washing. Taken together, these results suggest that VTN62-292 can provide a suitable and potentially improved animal-free substrate for the adhesion and culture of iPSCs.

### 3.3. Long Term Growth of iPSCs on VTN62-292

**Figure 2:**
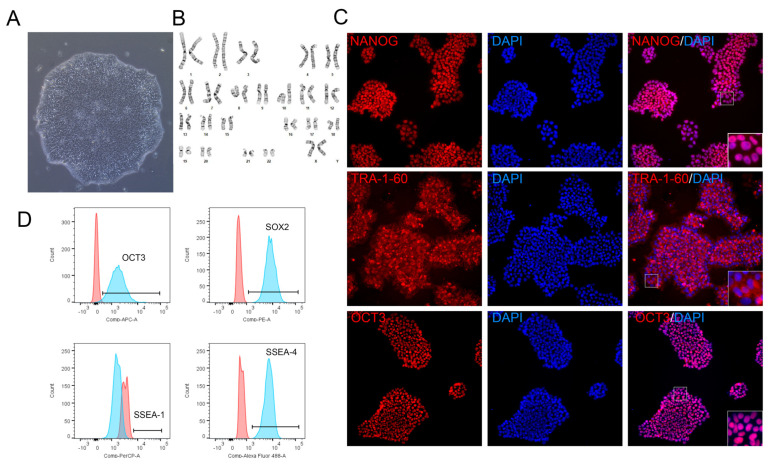
iPSCs maintain healthy morphology, karyotype, and stemness markers after long-term culture on VTN62-292. Brightfield image of iPSCs (BXS0114) cultured on VTN62-292 for over 13 passages show normal iPSC colony morphology, including defined borders and compact cells with large nucleus-to-cytoplasm ratio. Image is a 20× magnification. (**A**). G-band analysis of iPSCs (BXS0114) grown on VTN62-292 for over 13 passages show no chromosome abnormalities (**B**). iPSCs expanded on VTN62-292 for 15 passages were positive for the stemness markers NANOG, OCT3/4, and TRA1-60, as shown with immunofluorescence. Images were all taken at 20X magnification. (**C**). Insets are magnified images of the indicated region to show distinct nuclear localization of NANOG and OCT3 with more diffuse staining of surface protein TRA1-60 (**C**). After 15 passages, a different iPSC line (12-10a) expanded on VTN62-292 shows high expression of the stemness markers OCT3, SSEA-4, and SOX2 and no expression of SSEA-1 with flow cytometry (blue indicates antibody-stained cells, red indicates isotype) (**D**).

**Figure 3:**
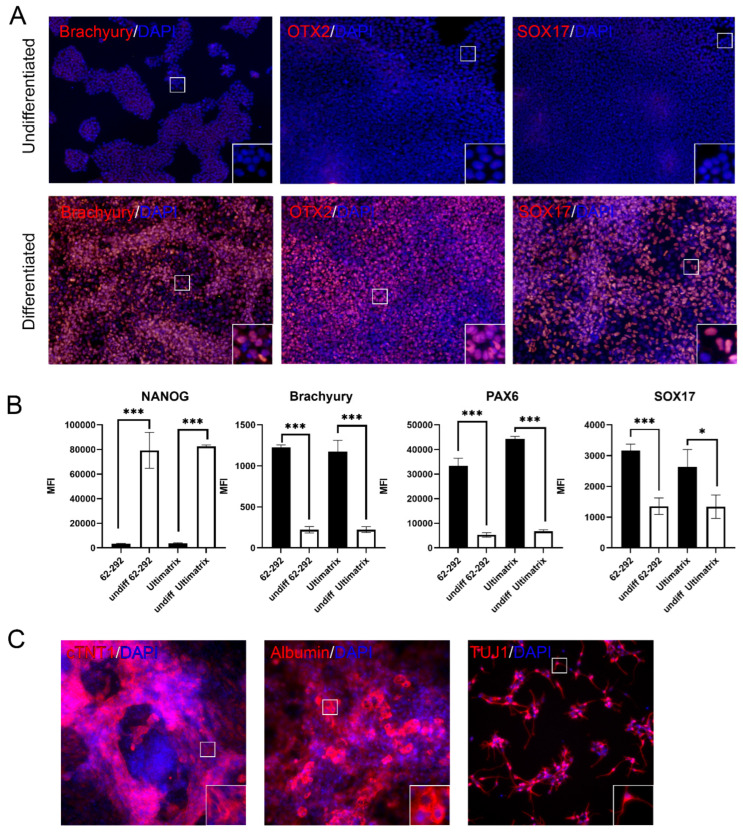
iPSCs cultured on VTN62-292 maintain pluripotency as demonstrated by differentiation potential. After long-term culture (over 10 passages) on VTN62-292, iPSCs (BXS0114) cultured on VTN62-292 develop into all three germ layers using in vitro differentiation protocols. Immunocytochemistry of trilineage differentiation shows increased expression of Brachyury (mesoderm), Otx2 (ectoderm), and SOX17 (endoderm) compared to undifferentiated controls. Images taken at 10× magnification. (**A**). Insets are magnified views of the indicated regions for each panel, showing the highly increased expression of these markers in the nuclei of most of the differentiated cells (**A**). Flow cytometry analysis confirms the differentiation potential of iPSCs (BXS0114) cultured on VTN62-292, with significant increases in germ-layer specific marker expression compared to undifferentiated controls. The change in marker expression is comparable to the same iPSCs cultured on Ultimatrix (Basement Membrane Extract, a.k.a. Matrigel) (**B**). Graphs of the mean fluorescent intensity (MFI) of differentiated cells compared to naïve iPSCs show appropriate loss of expression of NANOG and increased expression of Brachyury (following mesoderm differentiation), increased expression of PAX6 (following ectoderm differentiation), and increased expression of SOX17 (following endoderm differentiation) (N = 3) (**B**). Error bars indicate standard deviation. * *p* ≤ 0.05, *** *p* ≤ 0.001. piPSCs cultured on VTN62-292 differentiate into mature cell types of all three germ layers following growth factor-based differentiation protocols (**C**). Cardiomyocyte differentiation was detected with cardiac troponin T (cTNT1) expression, derivation of hepatocytes was detected with the transcription factor Albumin, and forebrain neurons were identified with beta III tubulin expression (TUJ1). Images taken at 20× magnification. (**C**). Insets are magnified views of the indicated regions for each panel showing localization of these markers in individual cells (**C**).

To assess the impact of vitronectin fragments on long-term iPSC culture, we investigated their effects on key cell quality attributes: growth rate, colony morphology, stemness markers, and pluripotency. Culture of iPSCs was performed with animal component-free iPSCs media; thus, with VTN62-292 the conditions were completely free of animal components. Multiple cell lines, including 12-10a, BXS0114, and BYS0110, were used to confirm the broad compatibility of the VTN fragments with different iPSC lines. Human iPSC colonies grown on VTN fragments showed morphology typical of healthy iPSC colonies. Specifically, the iPSC colonies showed tight cell clustering, large ratio of the cell nucleus to cytoplasm, and well-defined colony borders (Figure 2A). The morphology of colonies on VTN62-292 was maintained over multiple passages and was comparable to those grown on full length VTN, VTN-N, and Ultimatrix, remaining typical of iPSC colonies (Figure 3A; Supplemental Figure S3). Interestingly, iPSC colonies grew slightly faster on VTN62-292 compared to full-length VTN (40% confluent vs. 30% after 65 h of culture). The shorter VTN fragments did not support long-term iPSC expansion, as there was significant cell death. Even though iPSC colonies showed faster expansion on VTN62-292 compared to full-length VTN, VTN62-292 iPSCs showed a normal karyotype after more than 13 passages (Figure 2B). Immunocytochemistry (ICC) and flow cytometry were performed to confirm that iPSCs expanded on VTN62-292 retain positive expression of stemness markers. ICC images of iPSCs on VTN62-292 showed positive expression of the nuclear stemness markers OCT3 and NANOG as well as the surface marker TRA-1-60. Flow cytometry showed high expression (>95%) of OCT3, SOX2, and SSEA-4 without detecting the differentiation marker (SSEA-1). We also observed that continuous cell culture of iPSCs on VTN62-292 under animal-free conditions for over 44 passages resulted in cell colonies with normal morphology and stemness markers.

### 3.4. Maintenance of Pluripotency on VTN62-292

To assess pluripotency over long-term culture, human iPSCs were cultured on VTN62-292 and then differentiated into cells of the three primary germ layers (endoderm, mesoderm, and ectoderm) as well as neurons, hepatocytes, and cardiomyocytes. ICC showed that human iPSCs expanded on VTN62-292 retained their pluripotency and successfully differentiated to cells of the three germ layers. Differentiated cells showed positive expression of Brachyury (mesoderm), SOX17 (endoderm), and Otx2 (ectoderm), while undifferentiated cells did not show expression of these markers (Figure 3A). Flow cytometry confirmed these results, as mesoderm differentiated cells on VTN62-292 showed significantly increased expression of Brachyury with a concomitant decrease in NANOG (Figure 3B). Flow analysis of ectoderm differentiated cells showed increased expression of PAX6, while endoderm differentiated cells showed significantly increased expression of SOX17. Levels of these trilineage markers was similar between iPSCs differentiated on VTN62-292 compared to Ultimatrix (Figure 3B). Further analysis based on thresholding flow fluorescent intensity showed that after the mesoderm differentiation protocol iPSCs differentiated on VTN62-292 and Ultimatrix had less than 3% of cells expressing NANOG, while over 90% of undifferentiated cells were NANOG-positive (Supplemental Figure S3). In the same mesoderm protocol, over 85% of iPSCs differentiated on both VTN62-292 and Ultimatrix were Brachyury-positive, compared to less than 1% of naïve iPSCs. In a similar manner, irrespective of the substratum, ectoderm and endoderm differentiated cells showed over 80% of PAX6 and SOX17 expression, respectively, while less than 1% of the undifferentiated cells showed expression of these markers (Supplemental Figure S3). Additionally, the ICC data show that iPSCs grown on VTN62-292 were able to develop into terminally differentiated cells of all three germ layers, showing relevant biomarkers: cardiac troponin (cTNT1) for cardiomyocytes, albumin for hepatocytes, and β-III tubulin (or TUJ1) 1 for neurons (Figure 3C). In addition, we visually confirmed the robust differentiation of iPSCs into cardiac “sheets”, as indicated by the coordinated beating pattern of adjoined cardiomyocytes (Supplemental Video S2). Taken together, these data indicate that iPSCs grown under animal component-free conditions on VTN62-292 over long periods maintained cellular health and pluripotency.

### 3.5. Animal-Free Differentiation of iPSCs into Neurons with VTN62-292

**Figure 4:**
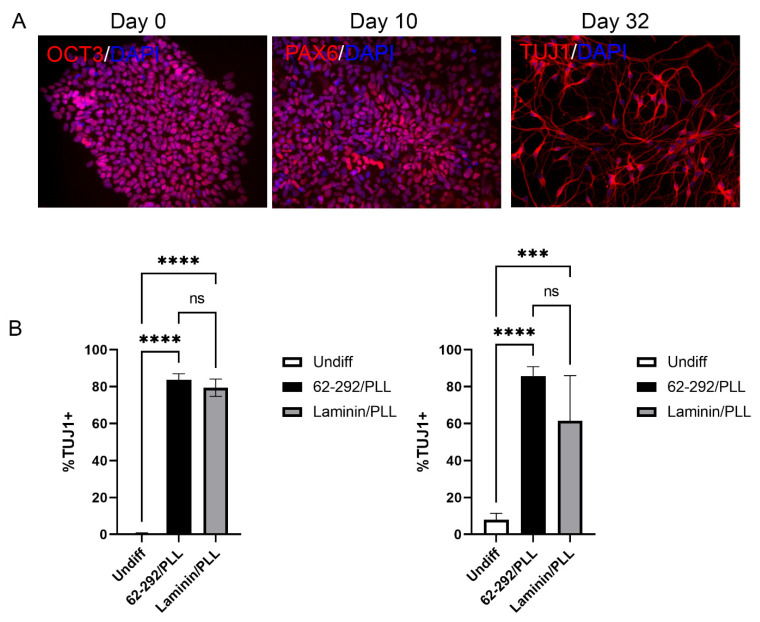
Differentiation of iPSCs into cortical neurons with VTN62-292 under defined animal-free conditions. iPSCs were differentiated on VTN62-292 into neural progenitor cells (NPCs) and subsequently into forebrain (cortical) neurons using a dual SMAD inhibition approach with reagents that are completely devoid of animal-containing materials (and also contain no human-derived materials). The complete workflow, from naïve iPSC culture through NPC generation and neuronal derivation, was performed on VTN62-292. Immunofluorescence was used to show the progression from naive iPSCs at day 0 (OCT3 expression) to NPCs at day 10 (PAX6 expression) and terminal differentiation into cortical neurons at day 32 (TUJ1 expression). Images were taken at 20× magnification (**A**). Quantification of the percentage of TUJ1-positive cells following neuronal differentiation from two different iPSC lines (BXS0114, left) and (12-10a, right) shows that the efficiency of neuronal derivation on VTN62-292 is comparable to laminin, a standard substrate for neuronal differentiation (N = 3) (**B**). Over 80% of the cells differentiated into neurons for both cell lines. Error bars indicate standard deviation. *** *p* ≤ 0.001, **** *p* ≤ 0.0001.

As cell therapies move into the clinic, the need for more defined and even animal-free cell culture conditions is important to improving the safety profile and reproducibility of these workflows. While xeno-free conditions have been established for the differentiation of forebrain neurons [43], no work has shown the derivation of neurons under bona fide animal-free conditions. To this end, we assessed whether we could generate highly pure forebrain neurons using a modified dual SMAD inhibition approach with all animal-free reagents, including cell culture media, supplements, small molecules, and VTN62-292 (Figure 4; see Section 2 for details). We first assessed the potential of iPSCs to produce neural progenitor cells (NPCs), and saw a dramatic decrease in the expression of OCT3/4 and a concomitant increase in PAX6 positive NPCs at day 10 in the differentiation protocol (Figure 4A). Following the passaging of the NPCs onto VTN92-292 together with poly-l-lysine (PLL) and continuation of the differentiation until day 32, we observed that the majority of cells had long β-III-positive neurites (TUJ1, (Figure 4A,B). In two separate iPSC lines, VTN62-292/PLL was as effective at generating and maintaining neurons as laminin/PLL, a typical substrate for neuronal cell culture (Figure 4B).

### 3.6. Reprogramming of PBMCs into iPSCs on VTN62-292

**Figure 5:**
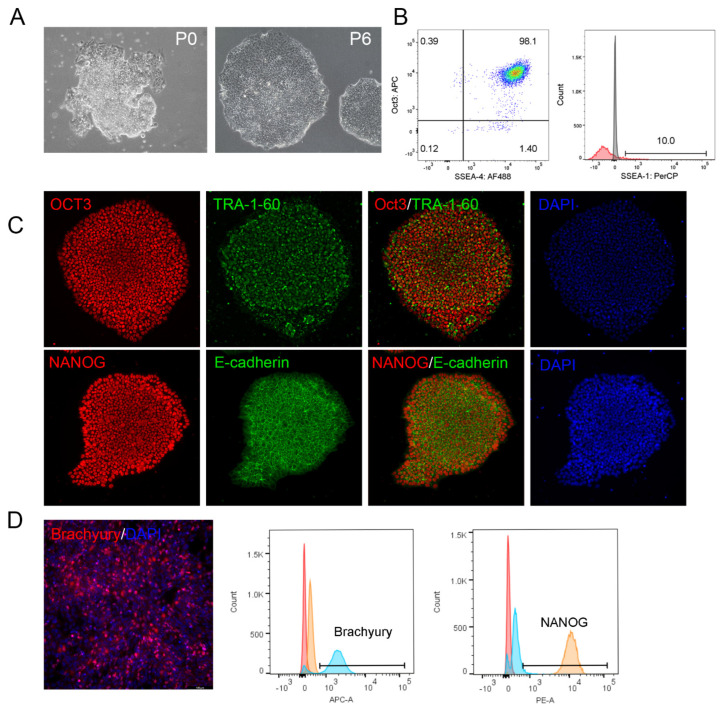
VTN62-292 supports the reprogramming of Peripheral Blood Mononuclear cells (PBMCs) into induced pluripotent stem cells (iPSCs). PBMCs were transduced with non-integrative Sendai viruses expressing the reprogramming factors OCT4, SOX2, KLF4, and cMyc. After 3 days in suspension, the cells were plated on VTN62-292, transitioned into ExCellerate iPSC medium, and allowed to grow for 21 days. Colonies were subcloned and propagated on VTN62-292. Brightfield images show the formation of nascent iPSC colonies before picking and subcloning (left, P0) and after the clone was expanded and passaged six times (right, P6). Images were taken at 20× magnification (**A**). Note that at P6 the iPSCs display the typical morphological features of PSCs, with tightly packed cells forming colonies with smooth borders. Flow cytometry confirmed the high expression of OCT3 and an additional PSC marker, SSEA-4, whereas the expression of SSEA-1 (red, isotype in gray), a marker of differentiation, was very low (**B**). Immunocytochemistry shows that the iPSC colonies express high levels of the pluripotency transcription factors OCT3/4 and NANOG in DAPI-positive nuclei, as well as other markers such as TRA 1–60 and E-Cadherin. Images were taken at 20× magnification (**C**). Following long-term cell culture and multiple passages (P17), the iPSCs were successfully differentiated into mesoderm, as indicated by increased expression of Brachyury from immunofluorescence images (taken at 20× magnification) and flow cytometry (**D**). The graphs generated from the flow cytometry experiments show high expression of the pluripotency maker NANOG in naïve cells (right plot, yellow), which also have low expression of brachyury (left plot, yellow). Following differentiation of the iPSCs into mesoderm, the cells exhibit low NANOG expression (left plot, blue) and high expression of Brachyury (left plot, blue) (**D**). These flow cytometry graphs indicate that undifferentiated iPSCs (yellow) show high levels of NANOG and low levels of Brachyury, while mesoderm differentiated cells (blue) show low levels of NANOG and high levels of brachyury. Red indicates cells stained with isotype antibody.

In previous work, reprogramming somatic cells on VTN-N facilitated the formation and propagation of nascent iPSC colonies under xeno-free conditions using episomal vectors [32]. While some commercial VTN fragments may support reprogramming, other work has failed to generate iPSCs on VTN with Sendai virus reprogramming strategies, which works well on Matrigel and Laminin substrates [47]. In light of our previous results showing improved growth of PSCs with the VTN62-292 fragment compared to other VTN variants, we wanted to evaluate the possibility of using this novel VTN variant to support reprograming using animal-free reagents. Using the Sendai-mediated introduction of the canonical Yamanaka factors (OCT 3/4, KLF4, SOX2, and c-MYC), we were able to successfully reprogram peripheral blood monocytes (PBMCs) into iPSCs (Figure 5). While using Ultimatrix (BME) as a substrate had slightly better efficiency, dozens of colonies formed with good morphology on VTN62-292 before passaging (P0), which were successfully subcloned and grew into typical iPSC colonies composed of tightly packed stem cells with large nuclei (Figure 5A). We observed that by P6 the newly subcloned iPSCs lines were propagating on VTN62-292, and expressed the stemness markers OCT3/4 and SSEA-4 but not the differentiated surface marker SSEA-1, as shown by flow cytometry (Figure 5B). ICC experiments confirmed the high expression of OCT3, NANOG, TRA-1-60, and E-cadherin after long-term cell culture of the iPSCs derived on VTN62-292 (Figure 5C). After continued expansion and passaging under animal component-free conditions, these iPSCs maintained good morphology at P17 with the expression of stemness markers, and were able to differentiate into downstream lineages such as mesoderm (Figure 5D). These results indicate that the VTN62-292 variant can be used as an animal-free substrate for reprogramming somatic cells into iPSCs.

Overall, we show that our animal-free vitronectin fragment, VTN62-292, supports the core of iPSC work: improved iPSC culture, multiple differentiations representing all three germ layers, and reprogramming. Our development of VTN62-292 is significant not only due to its increased yields, purity during manufacturing, and support of an increased iPSC growth rate but also due its ability to support animal-free forebrain neuron differentiation and reprogramming on vitronectin.

## 4. Discussion

Defined and scalable animal-free substrates are critical for ensuring experimental reproducibility and ease of transition to the clinic for developing iPSC cell therapies. Traditional iPSC culture substrates such as BME suffer from lot-to-lot variability and undefined physical and chemical characteristics. Over the past decade, defined substrates including full-length and truncated vitronectin have been introduced as alternatives to BME [32,48]. While truncated VTN has been made commercially available and at times shown better cell adhesion and viability than full-length VTN, its manufacturing is inefficient, as it requires further processing steps to deal with the accumulation of proteins in inclusion bodies [40].

In this work, a shorter VTN fragment, VTN62-292, was made in an animal-free process with greater yield and purity than possible with full-length or previously reported VTN truncations. While some commercially available truncated VTN fragments are available, such as VTN20-398 and VTN-N (VTN62-478), longer fragments up to amino acid 398 showed lower yields in our hands compared to VTN62-292. Exclusion of disulfide bonds could potentially contribute to this increased yield and purification. The production of proteins with disulfide bonds is difficult due to the reducing environment within the cytoplasm of *E. coli*; disulfide bond formation can be limited, and a protein may need to be exported to the oxidizing periplasm for proper formation [49,50]. Cysteine 293 of full-length VTN may form a disulfide bond with other cysteines, such as residue 430 [51]. VTN62-292 is truncated at residue 292, thereby excluding this disulfide bond formation. We propose that due to the difficulty of forming this disulfide bond in an *E. coli* system, its exclusion leads to increased manufacturability. The smaller VTN62-155 and VTN62-120 fragments, which would similarly exclude this disulfide bond formation, also showed increased manufacturability; however, these variants had worse performance in terms of iPSC cell adhesion and expansion. At higher concentrations these fragments may support limited iPSCs expansion, as suggested previously [39].

VTN62-292 is fully functional for iPSC cell culture, supporting cell growth and cell adhesion as well or better than full-length and VTN-N while maintaining normal karyotype. In fact, iPSCs grown on VTN62-292 showed a small but significant increase in growth compared to full-length vitronectin and similar growth compared to VTN-N. The effect was more pronounced at lower coating concentrations, with VTN62-292 showing much greater growth compared to iPSCs grown on full-length VTN and VTN-N. VTN62-292 maintains stemness and pluripotency, as shown by differentiation into all three germ layers and mature cell types such as cortical neurons, hepatocytes, and cardiomyocytes. Notably, VTN62-292 supports animal-free differentiation of iPSCs to forebrain neurons, which has not been previously reported. Importantly, VTN62-292 fully supports reprogramming of PBMCs into iPSCs, an essential step in producing iPSCs from patients and donors for research, characterization, and clinical use. Thus, our highly manufacturable VTN62-292 supports a fully defined and animal-free system for regenerative medicine and cell and gene therapy, including reprogramming, expansion, and differentiation.

More reproducible large-scale production of VTN62-292 could propel the advancement of various cell and gene therapy applications. Since the introduction of iPSCs in 2006, the use of iPSCs in basic and clinical research has continually increased [52,53] with a wide range of applications. For example, iPSCs can be differentiated into iT-cells or iNKs and utilized as part of immunotherapy to target cancer in clinical programs, or can be reprogrammed from patient cells to characterize their specific mutations as part of basic research. These advances have paved the way for the utilization of iPSCs to treat diseases from Parkinson’s and cancer to diabetes and Epidermolysis Bullosa. Large-scale production of iPSCs or iPSC-derived cells to treat these diseases could be supported by highly manufacturable substrates such as VTN62-292. Importantly, because VTN62-292 can be produced in large quantities, is well defined chemically, and can be produced in animal-free manner, it is highly amenable to cGMP clinical usage; for example, Allele biotechnology has adopted a full cGMP protocol, from reprogramming patient cells into iPSCs to expansion and cell banking, using recombinant vitronectin as the substrate for iPSC growth [54].

One key advantage of BME over an animal-free recombinant protein such as VTN62-292 is its use for 3D cell culture. Physiological conditions can behave dramatically differently in 3D; for example, breast epithelial cells develop similarly to tumor cells in 2D culture, but revert to normal growth in 3D culture. The use of 3D cell culture could both increase the success rate of drug discovery and reduce the number of animals needed for research [55,56]. However, unlike BME, vitronectin and many other ECM proteins can neither form 3D matrices by themselves nor recapitulate the appropriate growth factors to effectively support 3D cell cultures [57]. A possible alternative to BME is the development of synthetic matrices or hydrogels with the appropriate stiffness and binding sites to support organoid growth. Recent work has explored the linkage of smaller peptides, such as the RGD binding site of vitronectin, to enhance cell adhesion to these hydrogels [46,58]. We wonder how the linkage of VTN62-292, a larger but still small peptide compared to the size of full-length vitronectin, could influence cell growth and adhesion in a hydrogel-based 3D culture system. This would be a further step in developing a fully animal-free and defined 3D environment for all iPSC cultures.

## 5. Conclusions

Here, we demonstrate several advantages of VTN62-292: improved animal-free manufacturability; improved performance over other VTN variants, especially at lower concentrations; and enabling complete animal component-free iPSC workflows, including somatic cell reprogramming and neuronal differentiations. Taken together, VTN62-292 provides a key component that can support clinical translation of iPSC-based cellular therapies.

## 6. Patents

U.S. Provisional Patent Application No. 63/239,456 resulted from work described in this publication.

## Data Availability

All data are presented in the manuscript. Please contact authors for additional information.

## Supplementary Materials

The following supporting information can be downloaded at: https://www.mdpi.com/article/10.3390/cells13181566/s1, Figure S1: The vitronectin variants display different expression characteristics in animal-free bacterial expression systems. Figure S2: VTN62-292 supports the improved expansion of iPSCs at a low concentration. Figure S3: iPSCs differentiate readily into the three germ layers on VTN62-292. Video S1: VTN62-292 supports iPSC colony growth over time. Video S2: VTN62-292 supports long-term iPSC pluripotency as indicated by cardiomyocyte differentiation potential.
