# Supplementary material for: A Novel Recombinant Vitronectin Variant Supports the Expansion and Differentiation of Pluripotent Stem Cells in Defined Animal-Free Workflows
Source: Cells. 2024 Sep 17;13(18):1566. doi: 10.3390/cells13181566 (PMC11429963; doi:10.3390/cells13181566)
Supplement: Supplementary file 1 [file cells-13-01566-s001.zip › cells-3078432-supplementary.pdf]

**Appendix B Supplemental Data**

A

| VTN Construct | Relative Protein Yield | Soluble Protein Recovered? |
|---|---|---|
| 20-478 | 37.3 | NO |
| 62-478 | 29.5 | YES |
| 62-398 | 50.9 | YES |
| 62-292 | **112.7** | YES |
| 62-155 | 84.7 | YES |
| 62-120 | 166.7 | YES |

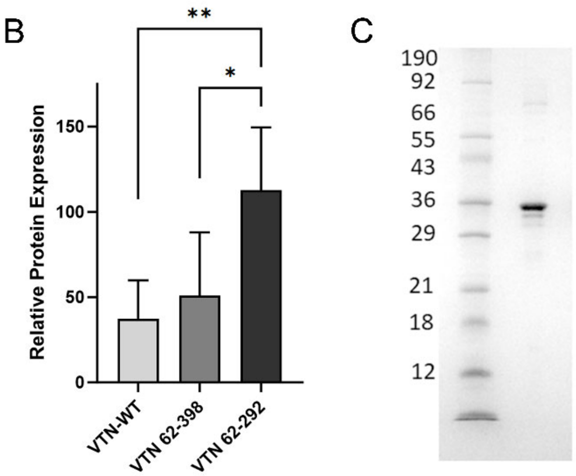


**Supplemental Figure 1: The Vitronectin Variants Display different expression characteristics in animal-free bacterial expression systems.**

Table of the relative yields and solubility from of the different VTN constructs screened in this work from one example purification experiment (**A**). Note that except for VTN 20-478, all the VTN variants had protein recovered in the soluble portion of the fermentation culture (**A**). VTN 62-120 has the highest soluble expression, but it displays poor performance in cell culture experiments. The quantification of five different purifications of VTN-WT (20-478), VTN 62-398, and VTN 62-292 shows the increased expression of VTN 62-292 in the soluble fraction compared to these other constructs (**B**). Error bars indicate standard deviation. *$p \leq 0.05$, **$p \leq 0.01$.

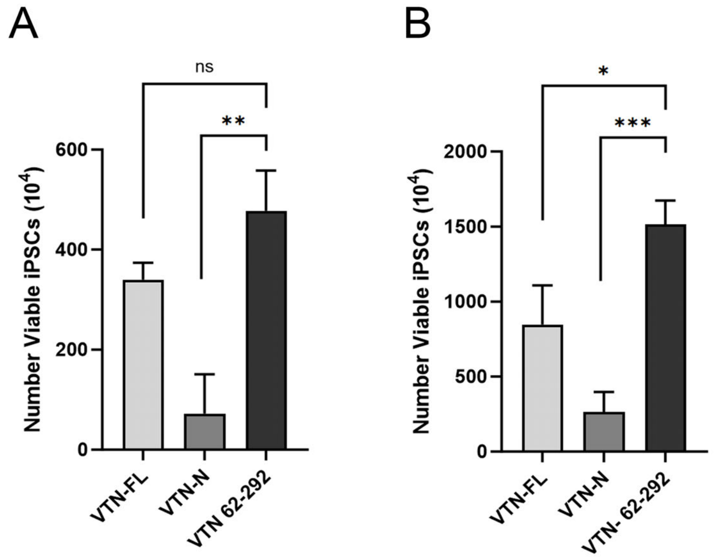


**Supplemental Figure 2: VTN 62-292 supports the improved expansion of iPSCs at a low concentration.** Two iPSC lines were seeded at the same density and cultured on VTN-FL, VTN-N and VTN 62-292 at 1 μg/mL for 3 days. The number of viable cells were recovered and quantified. The graph of the number of viable cells from BSX0114 iPSCs (**A**) and BYS110 cells (**B**) shows VTN62-292 supported iPSC cell cultures better than VTN-N at this low concentration (**A, B**). Error bars indicate standard deviation. *p≤0.05, **p≤0.01, ***p≤0.001.

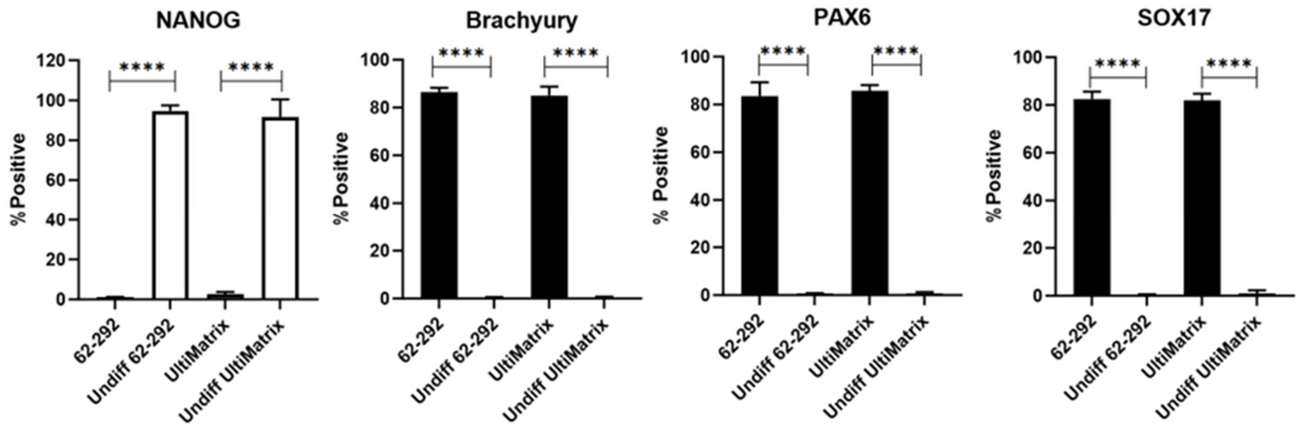


**Supplemental Figure 3: iPSCs differentiate readily into the three germ layers on VTN62-292.** Flow cytometry analysis based on thresholding from isotype controls shows the differentiation potential of iPSCs (BXS0114) cultured on VTN 62-292 and Ultimatrix with significant increases in the percent (%) of cells expressing germ-layer specific markers compared to undifferentiated controls. The change in the percentage of cells expressing the specific markers is comparable to the same iPSCs cultured on Ultimatrix (Basement Membrane Extract, a.k.a. Matrigel). The graphs of the percentage of differentiated cells compared to naïve iPSCs show a loss of expression of NANOG (from >90% to < 3%) and increased expression of Brachyury (from <1% to >80%) following mesoderm differentiation. Following ectoderm differentiation, the percentage of cells expressing PAX6 increases to over 80% compared to less than 1% on both VTN62-292 and Ultimatrix. Following endoderm differentiation, the percentage of cells expressing SOX17 increases to over 80% compared to less than 1% on both VTN62-292 and Ultimatrix. N=3 for all experiments. Error bars indicate standard deviation.**** p≤0.0001.

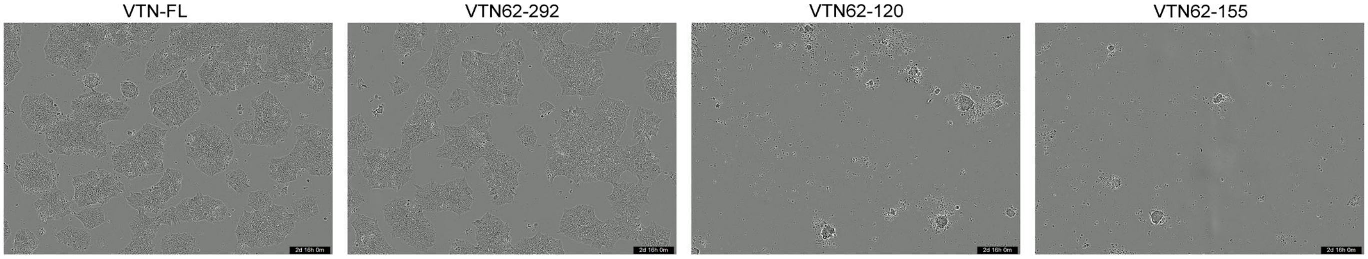


iPSC growth on VTN variants

**Supplemental Video 1: VTN62-292 supports iPSC colony growth over time.**

Live Cell imaging was performed to visualize iPSC cell growth on different VTN variants using an Incucyte® Live-Cell Analysis system (Sartorius, Gottingen, Germany). In these experiments, 12,000 cells/ cm2 were plated in 24 well plates and imaged every 60 minutes for over 2 days. Note that VTN62-292 supports the survival and proliferation of iSPCs in a manner similar to VTN-FL. Conversely, VTN62-120 and VTN62-155 appear to support initial cell attachment, but the iPSCs detach and appear to undergo apoptosis over time.

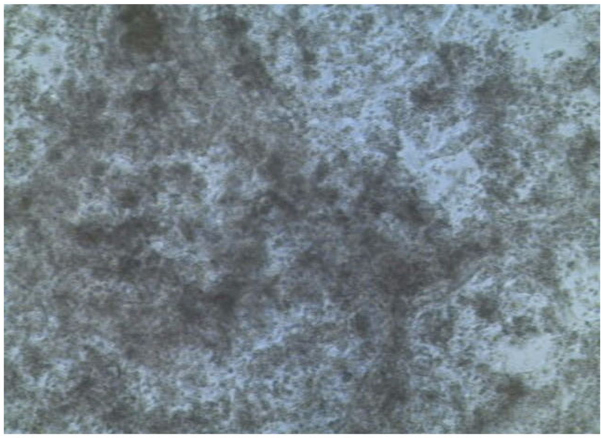
Day 11 Cardiomyocyte Differentiation

**Supplemental Video 2: VTN62-292 supports long-term iPSC pluripotency as indicated by cardiomyocyte differentiation potential.**

Live Cell imaging was performed to visualize cardiomyocytes differentiated from iPSCs that were maintained on VTN62-292. This video was collected 11 days after the initiation of the cardiomyocyte differentiation protocol (using the StemXVivo Cardiomyocyte kit from R&D Systems). Note that the cardiomyocytes form a continuous sheet of cells that exhibit clear synchronous contraction movements.
